# Expression and clinical significance of Cathepsin K and MMPs in invasive non-functioning pituitary adenomas

**DOI:** 10.3389/fonc.2022.901647

**Published:** 2022-08-16

**Authors:** Hongyan Liu, Saichun Zhang, Ting Wu, Zhaohui Lv, Jianming Ba, Weijun Gu, Yiming Mu

**Affiliations:** ^1^The Chinese PLA Medical School, Beijing, China; ^2^Department of Endocrinology, The First Medical Center of Chinese PLA General Hospital, Beijing, China

**Keywords:** Cathepsin K, sphenoid sinus, pituitary adenomas, invasion

## Abstract

**Background:**

Cathepsin K (CTSK) is a protease that degrades type I collagen and extracellular matrix, thereby contributing to bone resorption and tumor invasion. Some pituitary adenomas (PAs) could invade the sphenoid sinus (SS) and cavernous sinus (CS).

**Purpose:**

This retrospective cohort study aimed to study the expression of tumoral biomarkers (CTSK, MMP9, MMP2, TIMP2, and PTTG1) and evaluate their clinical significance in non-functioning pituitary adenomas (NFPAs) with different invasion patterns.

**Methods:**

We assessed the expression levels of candidate invasion-specific protein biomarkers CTSK, MMP9, MMP2, TIMP2, and PTTG1 by immunohistochemical staining in paraffin-embedded NFPA tumor tissues. Variations in staining intensity were analyzed in cases with SS and CS invasion and non-invasive NFPAs.

**Results:**

We found that the levels of CTSK were higher in PA cases with SS invasion than that in PA cases with CS invasion (95.57 ± 31.57 vs. 65.29 ± 29.64, P < 0.001), and the expression of MMP9 and MMP2 was higher in CS-invasive cases than that in SS-invasive cases (145.02 ± 49.25 vs. 111.80 ± 51.37, P = 0.002, and 138.67 ± 52.06 vs. 108.30 ± 41.70, P = 0.002). Multiple Cox regression demonstrated that higher CTSK expression (P=0.011), subtotal resection (P<0.001), invasion (P=0.037), and larger tumor diameter (P=0.001) were independent risk factors for recurrence. A positive correlation was observed between CTSK expression and tumor size (r=0.671, p<0.001). There was no significant difference in TIMP2 and PTTG1 levels between CS-and SS-invasive cases (97.42± 39.80 vs. 102.10± 43.22, P = 0.58 and 13.89 ± 4.59 vs. 12.56 ± 3.96, P = 0.14).

**Conclusion:**

Our data indicated that CTSK has the potential as a marker for SS invasion of PAs, whereas MMP9 and MMP2 may be markers for CS invasion. And CTSK may play an important role in tumor relapse.

## Introduction

Pituitary adenomas (PAs) are one of the most common intracranial tumors, with an overall community-based prevalence of 68-116 per 100 000 population (1–7), with the increasing annual incidence rate mainly due to incidentalomas. They are usually benign and slow-growing, causing symptoms because of excess hormone secretion or compression of close-by structures. However, they may become locally invasive, namely invasive PAs (IPAs), resulting in infiltration within the sphenoid sinus (SS; the destruction of the sellar floor is the first step in this process), cavernous sinus (CS), or suprasellar structures, especially nonfunctioning PAs (NFPAs) because of a lack of typical clinical symptom. The main clinical manifestations of NFPAs are compression effects, such as visual disturbance, headache, and hypopituitarism. It is difficult to achieve a total resection of the tumor tissues for IPA patients and they are associated with a higher rate of recurrence and a lower rate of remission.

Abundant articles have analyzed and hypothesized the mechanisms involved in the invasion process of PAs. Many studies have investigated the correlation between the invasion of PAs and several biological markers, including matrix metalloproteinase-9 (MMP9), matrix metalloproteinase-2 (MMP2), tissue inhibitor of metalloproteinases-2 (TIMP2), and pituitary tumor transforming gene 1 protein (PTTG1, also called securin) (8–13). Our previous meta-analysis has shown that MMP9 and MMP2 expression in IPAs was distinctly higher than that in noninvasive PAs (non-IPAs) (14). Chen, K. et al. reported that epithelial-mesenchymal transition (EMT)-related markers in serum exosomes were associated with invasion of PAs (15). However, the established mechanisms still need to be explored. Some researchers believe that PAs extending into CS are not invasive, and the extension into the CS occurs due to its medial wall weakness, and sometimes defect (10, 16). To our knowledge, no study has investigated the protein expression in SS invasion only or compared the expression levels in SS- and CS-invasive PAs.

Cathepsin K (CTSK), one of the papain-like cysteine proteases (17), is expressed predominantly in activated osteoclasts. CTSK, having high matrix-degrading activity, could degrade collagens, especially type I collagen (18), which is the main component (90%) of bone collagen fibers, and plays a crucial role in bone resorption. Beyond the osteoclasts, CTSK is highly expressed in various malignant tumors, such as breast carcinomas, lung cancers, melanomas, ovarian carcinomas, and prostate cancers (19–24). Besides, a higher level of CTSK expression was found in patients with bone metastasis or invasive tumor than that in patients with primary tumors (22, 25, 26). The bone metastases in breast carcinomas or prostate cancers are predominantly osteolytic (27, 28). *In vitro* experiments, animal models and clinical trials have evaluated the efficiency of several CTSK inhibitors (CatKi). *In vitro* experiments of Liang, W. et al. showed that CatKi reduced tumor invasion of prostate cancers and bone resorption induced by conditioned media of prostate cancers (29). They further conducted experiments in the animal models and found that CatKi prevented and reduced prostate cancer establishment and bone metastasis. Similarly, Le Gall et al. observed a reduction in osteolytic lesions in nude mice with bone metastatic breast cancer after being administered with CatKi AFG-495 (25). Moreover, a clinical randomized controlled trial (RCT) by Jensen et al. found that for breast cancer patients with bone metastases, the efficacy and safety of CatKi odanacatib were similar to that of zoledronic acid (ZOL), a standard therapeutic option for breast cancer bone metastases, after 4 weeks of treatment (30). Like bone metastases of breast carcinomas or prostate cancers, PAs with SS or clivus invasion lead to osteolytic lesions (31). Thus, we hypothesized that CTSK may play a role in SS or clivus invasion of PAs, which has been published previously (32). The present study aimed to verify our hypothesis, study the clinical significance of CTSK, and investigate the differential expression of MMP9, MMP2, TIMP2, and PTTG1 in SS and CS invasion of NFPAs.

## Materials and methods

### Patients

NFPAs were used in our study only because we could not determine whether the abnormal hormone secretion in functioning PAs will affect the expression of CTSK. Of the patients undergoing surgery for resection of PAs at the Chinese PLA General Hospital (Beijing, P.R. China) between 2011 and 2018, 176 cases met the following criteria: 1) transsphenoidal surgery; 2) no radiotherapy, chemotherapy, or any other medical interventions before the surgery; 3) complete clinical and radiological information in the database; 4) NFPAs. The diagnosis of the tumor and its functional status was according to its clinical, biochemical and radiological features, and was verified by immunohistological staining for prolactin (PRL), growth hormone (GH), adrenocorticotropic hormone (ACTH), thyroid-stimulating hormone (TSH), follicular stimulating hormone (FSH), and luteinizing hormone (LH). IHC criteria for NFPAs are silent gonatotropinomas and null-cell adenomas. The following criteria were used to define invasion: (1) preoperative images showed that Knosp/Hardy grades were 3 or 4; (2) intraoperative inspection of tumor infiltration within the SS (destruction of sellar floor or clivus only were included) and CS. The invasion was diagnosed only when both of the above criteria are met. 176 cases included 44 PAs invading SS only, 50 PAs invading CS only, 2 PAs invading both SS and CS, and 80 non-IPAs. Finally, Paraffin-embedded tissues of 174 patients including PAs invading SS only (n =44), PAs invading CV only (n = 50), and noninvasive PAs (n = 80) were used for immunohistochemical staining. In the SS invasion group, there were 20 women and 24 men, with a mean age of 50.2 ± 12.0 years (range 19-81 years). In the CS invasion group, there were 28 women and 22 men, with a mean age of 52.4 ± 14.8 years (range 19-78 years). In the non-invasion group, there were 40 women and 40 men, with a mean age of 48.1 ± 12.9 years (range 10-75 years). Micro-adenomas were defined as the largest tumor diameter (LTD) < 1cm and macro-adenomas were defined as the LTD ≥1cm, among which the LTD > 3cm were defined as large adenomas and the LTD > 4cm were defined as giant adenomas. There were 52 patients with large adenomas and 18 patients with giant adenomas.

### Postoperative follow-up

Postoperative telephone or outpatient follow-up was conducted every 6 months until December 2021. MR scans were used to evaluate tumor relapse. Tumor relapse is defined as tumor recurrence after gross-total resection (GTR) or an increase of residual tumor more than 2-mm in at least one dimension after subtotal resection (STR) according to contrast-enhanced (CE) T1WI (33). This study was approved by the Ethics Committee of the Chinese (People’s Liberation Army (PLA) General Hospital.

### Antibodies and dilutions

The following antibodies and kits were used: rabbit monoclonal primary antibody to human CTSK (ab207086, dilution 1:1,000), mouse monoclonal primary antibody to human MMP9 (ab58803, 1:500), mouse monoclonal primary antibody to human MMP2 (ab86607, 1:200), mouse monoclonal primary antibody to human TIMP2 (ab1828, 1:50), mouse monoclonal primary antibody to human PTTG1 (ab3305, 1:100), and mouse and rabbit specific HRP/DAB (ABC) detection IHC Kit (ab64264, including biotinylated goat anti-polyvalent, streptavidin peroxidase, 50×DAB chromogen, DAB substrate, and hydrogen peroxide block). All antibodies were purchased from Abcam (Cambridge, MA USA).

### Immunohistochemical staining procedure

Paraffin sections (4 μm-thick) were deparaffinized in xylene and rehydrated through graded ethanol (100% I, 100% II, 90%, and 80%). For antigen retrieval, the sections were heated at 100 °C in a microwave in 1× citrate buffer, pH 6.0 (MMP9, MMP2, and TIMP2 staining) or an EDTA solution, pH 9.0 (CTSK and PTTG1 staining) for 2.5 min. To block the endogenous peroxidase activity, the slides were immersed in 3% hydrogen peroxide (H_2_O_2_) in methanol for 15 min and then washed three times with 1× phosphate-buffered saline (PBS) for 5 min. The slides were then incubated with the primary antibodies indicated above, overnight at 4 °C in a humidified chamber, and then washed with PBS. Biotinylated goat anti-polyvalent and streptavidin peroxidase were then added and incubated for 10 min at 20-25°C, in succession. After washing with PBS, the slides were treated with 3,3’-diaminobenzidine (DAB) substrate for 15 min at 20-25°C. The slides were then washed two times with water, lightly counterstained with hematoxylin, and finally, mounted with coverslips and DPX after dehydration through an ethanol gradient (80%, 90%, 100% II, and 100% II) (34).

### Analysis of immunohistochemical results

For each case, 2 slides were analyzed. For each slide, 5 microscopic fields were counted. The intensity of expression was scored as 0 (negative staining), 1 (weak staining), 2 (moderate staining), or 3 (strong staining). The extent of expression was calculated using the Image Pro-Plus (IPP) software (Media Cybernetic, USA) (35).

For CTSK, MMP9, MMP2, and TIMP2, the final score was calculated as follows: (1×% of weak staining) + (2×% of moderate staining) + (3×% of strong staining). For PTTG1, the final score was calculated as follows: percentage of weak staining + percentage of moderate staining + percentage of strong staining (36). The expression of final score was described as numbers without %.

Taking the tertile 1 (Q1) value of expression as the boundary line, patients could be separated into high expression and low expression groups.

The assessment of protein expression was conducted independently by two pathologists. To constrain bias, both pathologists were blinded to whether the images were from invasive cases or non-invasive cases.

### Statistical analyses

Continuous variables are expressed as the mean ± standard deviation (SD) or median (inter-quartile range, IQR) (for data not conforming to normal distribution) and categorical variables are expressed as frequency and percentage. Continuous variables were compared using the Student’s T-test (for data conforming to normal distribution and homogeneity of variance) or Wilcoxon test or Kruskal-Wallis H test (for data not conforming to normal distribution or homogeneity of variance). Categorical variables were compared using the Chi-square test. Pearson analysis was used for correlation analysis of all possible pairwise combinations of CTSK, MMP9, MMP2, TIMP2, and PTTG1 proteins’ expression and correlation analysis of CTSK expression and tumor size. P < 0.05 was considered statistically significant. The Cox regression model was used to investigate factors related to recurrence of PAs. The SPSS software (version 25.0, IBM) and GraphPad Prism 8 (GraphPad Software Inc., San Diego, CA, USA) were used to perform all analyses.

## Results

### Expression and invasion

#### CTSK and invasion

**Figure 1:**
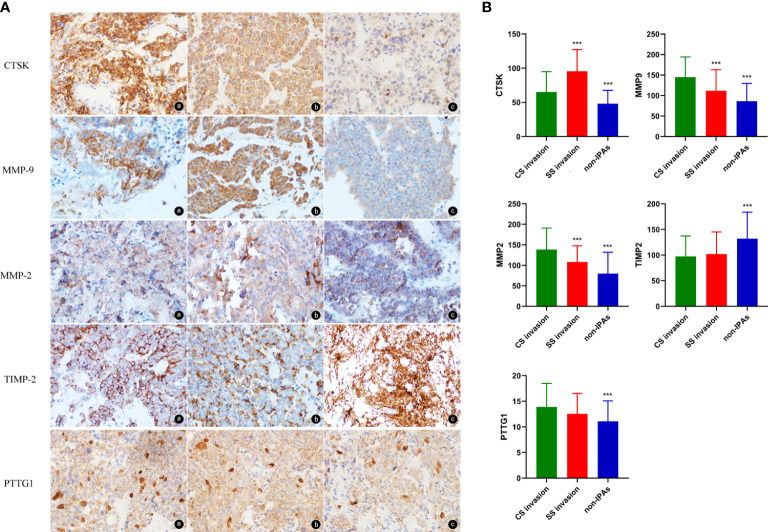
**(A)** Representative images of CTSK, MMP9, MMP2, TIMP2, and PTTG1 staining of PAs (magnification, 40×). a: PAs with SS invasion, b: PAs with CS invasion, c: non-IPAs. **(B)** Expression levels (%) (mean ± SD) of CTSK, MMP9, MMP2, TIMP2, and PTTG1 (Student’s t-test: ***p<0.001). SD: standard deviation.

Representative images of CTSK staining were showed in **Figure 1A**. CTSK was expressed in 91% of the PA cases with SS invasion, 78% of the PA cases with CS invasion, and 51% of the non-invasive PA cases. Additionally, CTSK expression was higher in invasive than in non-invasive PA cases (79.46 ± 33.98 vs. 48.09 ± 19.50, P < 0.001), and in SS-invasive cases than in CS-invasive cases (95.57 ± 31.57 vs. 65.29 ± 29.64, P < 0.001, **Figure 1B**).

#### MMP9 and invasion

MMP9 was expressed primarily in the cytoplasm (**Figure 1A**). MMP9 was expressed in 100% of the SS-invasive cases, 100% of the CS-invasive cases, and 85% of the non-invasive PA cases. MMP9 expression was higher in invasive cases than in non-invasive cases (129.47 ± 52.69 vs. 86.35 ± 43.44, P < 0.001), and in CS-invasive cases than in SS-invasive cases (145.02 ± 49.25 vs. 111.80 ± 51.37, P = 0.002, **Figure 1B**).

#### MMP2 and invasion

MMP2 was expressed primarily in the cytoplasm (**Figure 1A**). MMP2 was expressed in 100% of the SS-invasive cases, 100% of the CS-invasive cases, and 90% of the non-invasive cases. MMP2 levels were higher in invasive than in non-invasive PA cases (124.46 ± 50.11 vs. 79.99 ± 51.87, P < 0.001), and in CS-invasive cases than in SS-invasive cases (138.67 ± 52.06 vs. 108.30 ± 41.70, P = 0.002, **Figure 1B**).

#### TIMP2 and invasion

TIMP2 was expressed primarily in the cytoplasm (**Figure 1A**). TIMP2 expression was lower in invasive cases than in non-invasive cases (99.61 ± 41.28 vs. 132.15± 51.64, P < 0.001). There was no significant difference in TIMP2 levels between CS-and SS-invasive cases (97.42± 39.80 vs. 102.10± 43.22, P = 0.58, **Figure 1B**).

#### PTTG1 and invasion

PTTG1 was expressed both in cytoplasm and nuclei (**Figure 1A**). PTTG1 expression was higher in invasive than in non-invasive cases (13.27 ± 4.34 vs. 11.08 ± 4.00, P = 0.001). There was no significant difference in PTTG1 expression between CS- and SS-invasive cases (13.89 ± 4.59 vs. 12.56 ± 3.96, P = 0.14, **Figure 1B**).

### Expression and tumor size

**Figure 2:**
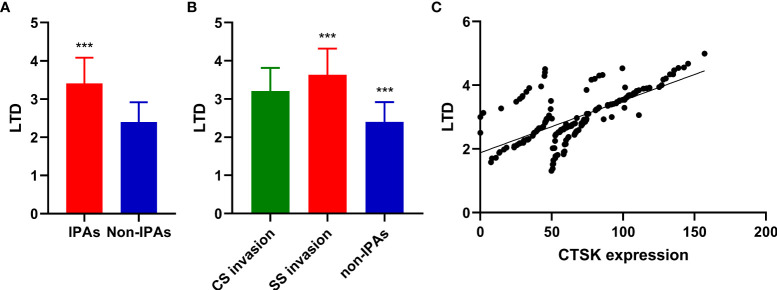
Correlations between PAs invasion, CTSK expression, and tumor size. **(A)**: Tumor size (cm) (mean ± SD) in in invasive and non-invasive PAs (Student’s t-test: ***p<0.001). **(B)** Tumor size (cm) (mean ± SD) in CS invasive PA, SS invasive PAs, and non-IPAs (Student’s t-test: ***p<0.001). **(C)** Correlations between CTSK expression (%) and tumor size (cm). (r=0.671, p<0.001). SD: standard deviation.

The mean LTD in invasive and non-invasive cases were 3.41 ± 0.67 cm and 2.40 ± 0.52 cm, respectively (p<0.001, **Figure 2A**). The mean LTD in SS invasive and CS invasive cases were 3.64 ± 0.68 cm and 3.21 ± 0.60 cm, respectively (p=0.002, **Figure 2B**).

The mean LTD in CTSK high-expression cases and CTSK low-expression cases were 3.04 ± 0.82 cm and 2.76 ± 0.69 cm, respectively (p=0.028). A positive correlation was observed between CTSK expression and tumor size (r=0.671, p<0.001) (**Figure 2C**). There was no significant difference in the mean LTD between different MMP9, MMP2, TIMP2, and PTTG1-expression groups (all p>0.05, Additional file 1).

### Expression and clinical features of patients

The postoperative follow-up time was 45.74 ± 20.33 months (range from 2.17 to 70.6 months). In total, 63 (36.2%) patients had tumor relapse and the average time to recurrence was 26.59 ± 11.24 months. 40 of 94 (42.6%) IPA patients recurred with an average time of 24.34 ± 9.91 months.

**Figure 3:**
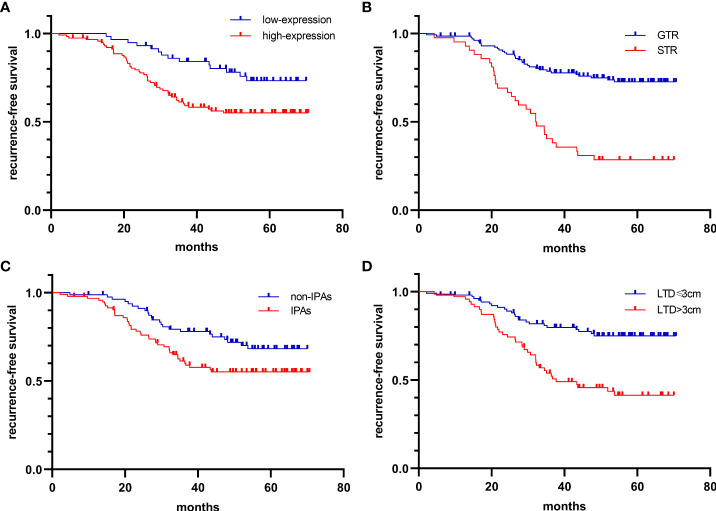
Kaplan-Meier curves for the recurrence-free interval. **(A)** high-CTSK expression vs. low-CTSK expression [HR: 2.31 (1.22-4.39), P=0.011], **(B)** GTR vs. STR [HR: 0.34 (0.21-0.57), P<0.001], **(C)** IPAs vs. Non-IPAs [HR: 0.65 (0.44-0.97), P=0.037], **(D)** LTD>3cm vs. LTD ≤ 3cm [HR: 3.60 (1.68-7.75), P=0.001].

The Cox regression model was used to further investigate the factors related to tumor relapse. Univariate Cox analysis demonstrated that the older [HR: 2.22 (1.22-4.02), p=0.009], higher CTSK expression [HR: 2.22 (1.22-4.02), p=0.009], STR [HR: 0.27 (0.16-0.44), p<0.001], invasion [HR: 1.32 (1.02-1.70), p=0.036], and larger LTD [HR: 2.87 (1.72-4.78), p<0.001] were associated with recurrence. Multiple Cox analysis demonstrated that higher CTSK expression [HR: 2.31 (1.22-4.39), P=0.011], STR [HR: 0.34 (0.21-0.57), P<0.001], invasion [HR: 0.65 (0.44-0.97), P=0.037], and larger LTD [HR: 3.60 (1.68-7.75), P=0.001] were independent risk factors for recurrence (**Table 1**). Kaplan–Meier curve demonstrated that higher CTSK expression, STR, invasion, and larger LTD group had worse recurrence-free survival (RFS) (**Figure 3**). Regarding the surgical resection degree, 42 (24.1%) patients had a residual tumor in the CTSK high-expression group while only 9 cases (5.2%) had a residual tumor in the low-expression group. However, this difference was not statistically significant (p=0.06). Compression symptoms are the main clinical manifestations of NFPAs. Patients with compression symptoms had a higher CTSK expression than those without (p=0.001). No evident correlation was found between expression levels of CTSK and other clinical features of PAs, including gender, age, pituitary apoplexy, and tumor texture. Detailed clinical features were summarized in **Table 2**. No evident correlation was found between expression levels of MMP9, MMP2, TIMP2, and PTTG1 and clinical features of PAs, including gender, age, compression symptoms, pituitary apoplexy, tumor texture, resection degree, and recurrence. Detailed clinical features were summarized in Additional file 2.

**Table 1:** Factors related to pituitary adenoma recurrence by Cox regression analysis.

	Univariate Cox regression		Multiple Cox regression	
	HR (95%)	P value	HR (95%)	P value
Age^1^	1.02 (1.00-1.04)	**0.038**	1.02 (0.99-1.04)	0.06
Sex^2^	1.03 (0.63-1.68)	0.920		
Tumor texture^3^	reference			
	3.23 (0.44-23.52)	0.247		
	3.45 (0.47-25.63)	0.225		
MMP9 expression^4^	0.98 (0.58-1.64)	0.930		
MMP2 expression^4^	1.22 (0.71-2.08)	0.477		
TIMP2 expression4	1.36 (0.79-2.35)	0.270		
PTTG1 expression^4^	1.29 (0.76-2.20)	0.339		
CTSK expression^4^	2.22 (1.22-4.02)	**0.009**	2.31 (1.22-4.39)	**0.011**
Resection degree^7^	0.27 (0.16-0.44)	**<0.001**	0.34 (0.21-0.57)	**<0.001**
Invasion^6^	1.32 (1.02-1.70)	**0.036**	0.65 (0.44-0.97)	**0.037**
LTD^5^	2.87 (1.72-4.78)	**<0.001**	3.60 (1.68-7.75)	**0.001**

^1^age as a continuous variable. ^2^male vs. female, ^3^cystic vs. solid vs. cystic & solid, ^4^high expression vs. low expression, ^5^>3cm vs. ≤3cm, ^6^IPAs vs. non-IPAs, ^7^GTR vs. STR. LTD, the largest tumor diameter; IPAs, invasive pituitary adenomas; non-IPAs, non-invasive pituitary adenomas. GTR, gross-total resection; STR, subtotal resection. The bold values mean p<0.05.

**Table 2:** Relationship between CTSK expression and clinical features of patients.

Clinical features	N	CTSK expression		X^2^	p
		High 116	Low 58		
Sex					
Male	86	54	32	1.15	0.284
Female	88	62	26		
Age (year)					
<50	90	59	31	0.104	0.748
≥50	84	57	27		
LTD					
≤3cm	104	62	42	5.784	**0.016**
>3cm	70	54	16		
Invasiveness					
IPAs	94	80	14	31.283	**0.000^#^**
SS-IPAs	44	42	2	6.988	0.008^*^
CS-IPAs	50	38	12		
non-IPAs	80	36	44		
Compression symptoms					
Yes	112	85	27	12.04	**0.001**
No	62	31	31		
Pituitary apoplexy					
Yes	30	18	12	0.725	0.395
No	144	98	46		
Tumor texture					
Cystic	7	3	4		
Solid	110	74	36	0.23	0.88
Cystic & solid	57	39	18		
Resection degree					
Total	132	83	49	3.531	0.06
Residual	42	33	9		
Recurrence					
Yes	63	49	14	5.486	**0.019**
No	111	67	44		

^#^P-value for comparison of IPAs and Non-IPAs, *P value of comparison of SS-IPAs and CS-IPAs. LTD, the largest tumor diameter; SS, sphenoid sinus; CS, cavernous sinus; IPAs, invasive pituitary adenomas; non-IPAs, non-invasive pituitary adenomas. The bold values mean p<0.05.

### Relationship between CTSK, MMP-9, MMP-2, TIMP-2, PTTG1 Protein expression

**Figure 4:**
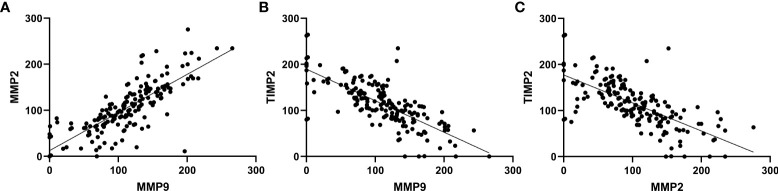
Correlations between expression of MMP9, MMP2 and TIMP2. **(A)** MMP9 and MMP2 (r=0.824, p<0.001). **(B)** MMP2 and TIMP2 (r=-0.700, p<0.001). **(C)** MMP9 and TIMP2 (r=-0.743, p<0.001).

A positive correlation was observed between MMP9 and MMP2 expression (r=0.824, p<0.001). There is a negative correlation between MMP9 and TIMP2 (r=-0.743, p<0.001) and MMP2 and TIMP2 (r=-0.700, p<0.001). No significant correlation was found between the expression of others (**Figure 4**).

## Discussion

To our knowledge, this is the first study that investigates the difference in the expression of MMP9, MMP2, TIMP2, and PTTG1 in PAs with SS and CS invasion and explores the expression of CTSK in SS invasion of PAs. Our results showed that PAs with SS invasion express significantly higher levels of CTSK compared with PAs with CS invasion and non-invasive controls, confirming our hypothesis. CTSK is expressed predominantly in osteoclasts, and can also be expressed in thyroid epithelial cells and skin fibroblasts (37). Christensen et al. have reported that CTSK, besides degrading type I collagen, can cleave and activate MMP9 in acidic environments such as those seen in tumors and during bone resorption (38), therefore contributing to the degradation of type I collagen and other matrix proteins and facilitating bone metastases. A similar bone resorption mechanism (*via* CTSK expression) might be involved in the SS invasion of PAs.

We also found that the higher CTSK expression was associated with higher rate of compression symptoms and large adenomas. It is well known that oversized tumors were responsible for compression symptoms of PAs. *In vitro* experiments of Yang, H. et al. demonstrated that CTSK high-expression contributed to the proliferation and migration of human non-small cell lung cancer (NSCLC) cells by activating the mammalian target of rapamycin (mTOR) signaling pathway (39). *In vitro* experiments of Gu, X. et al. found that down-regulation of CTSK expression could inhibit the proliferation and migration of breast cancer cells (40). Thus, we consider that CTSK may have a potential role in the proliferation of PA cells.

Our results show that IPA tissues express significantly higher levels of MMP9 and MMP2 compared with non-IPA tissues. Additionally, PA tissues with CS invasion express higher levels of MMP9 and MMP2 compared with PA tissues with SS invasion, indicating the important role of MMP9 and MMP2 in CS invasion. MMP9 and MMP2 degrade primarily collagen type IV, which is the prominent component of the basement membrane, pituitary capsule, medial wall of the cavernous sinus, and reticular fiber roof of the hypophysis (41), and it is also the key component of the dura mater. Notably, Deryugina et al. have found that MMP9 and MMP2 can contribute to tumor angiogenesis, which is important in tumor invasion and metastasis (42).

Our results show that IPA tissues express significantly higher levels of PTTG1 compared with non-IPA tissues. Malik *et al.* have reported that PTTG1 contributes to tumor growth and metastasis by increasing the expression of MMP-2 (43). Similarly, Lim et al. have found that PTTG1 can inhibit trophoblast invasion by regulating the expression and secretion of MMP9 and MMP2 (44). Liu et al. have found that folate receptor α (FRα)-targeted liposomes loaded with doxorubicin (F-L-DOX) have the anti-invasive ability in NFPAs by suppressing the secretion of MMP9 and MMP2 (45). Additionally, Barreiros et al. have reported that Toll-like receptor 2 (TLR2) and myeloid differentiation factor 88 (MyD88) are associated with apical periodontitis progression, possibly through the modulation of MMP9 and MMP2 (46). Besides, some proteins related to PA invasion, such as the discoidin domain receptor-1 (DDR1) (47), and β-catenin (48), have been reported to function by regulating the levels of MMP9 and MMP2. These data indicate that MMP9 and MMP2 play an important role in the invasion of PAs, particularly in CS invasion, and other related proteins may act through the regulation of MMP9 and MMP2 or in an MMP-independent manner.

We found that IPA tissues expressed significantly lower levels of TIMP2 compared with non-IPA tissues; this data differs from that obtained by Gültekin et al. (8). TIMP2 inhibits MMP activity by forming noncovalent complexes with the MMP protease active site. TIMP2 was initially shown to prohibit cell proliferation and migration *via* inhibiting the function of MMPs (49), and many studies found that TIMP2 could also be predictive of better prognosis in several cancer types, such as breast cancer (50), gastric cancer (51), colorectal cancer (52), and non-small cell lung cancer (49). However, TIMP2 can also act as an activator of MMPs, by forming the complex MT1-MMP/TIMP2 (53). Stetler-Stevenson *et al.* have found that TIMP2 can play other roles in an MMP-independent manner (54). Therefore, the function of TIMP2 is complex, and additional studies are needed to investigate the relationship between TIMP2 expression and tumor invasion.

Our results showed that the recurrence rate of NFPAs was 36.2% after surgical resection. Many articles have reported that larger LTD, invasion, and STR are related to a higher risk of recurrence (55–57). Similarly, a significant association of larger LTD, invasion, and STR, and with recurrence was observed in our study. We also found that higher CTSK expression, not MMP9, MMP2, TIMP2, and PTTG1 expression, was a risk factor for recurrence. Up to now, this is the first study to explore the relationship between CTSK expression and the recurrence of PAs. However, the relationship between CTSK expression and the recurrence of other cancers has been studied. Cordes, C. et al. reported that in patients with NSCLC, a high expression of CTSK was associated with a substantial increase of recurrence and mortality (21).

In conclusion, our data indicated that CTSK has the potential as a marker for SS invasion of PAs, whereas MMP9 and MMP2 may be markers for CS invasion. And CTSK may play an important role in tumor relapse. In the future, CTSK is expected to be as a predictor of bone invasion and recurrence of NFPAs in clinical practice. Our results provide insights to explore and understand the mechanisms involved in the invasion of PAs and seek new therapies for patients with IPAs. In the next future, we plan to perform studies on animal models, to investigate the function of CatKi in SS invasion of PAs.

## Data availability statement

The original contributions presented in the study are included in the article/**Supplementary Material**. Further inquiries can be directed to the corresponding author.

## Ethics statement

The studies involving human participants were reviewed and approved by Ethics Committee of the Chinese PLA General Hospital. The patients/participants provided their written informed consent to participate in this study.

## Author contributions

HL experimented and analyzed the data. She was a major contributor to writing the manuscript. SZ, WG, and TW supervised the histological examination. ZL and JB made substantial contributions to conception and design, or acquisition of data, or analysis and interpretation of data, and have been involved in revising it critically for important intellectual content. WG and YM were the superior advisors. They gave final approval of the version to be published and agreed to be accountable for all aspects of the work in ensuring that questions related to the accuracy or integrity of any part of the work are appropriately investigated and resolved. All authors read and approved the final manuscript.

## Acknowledgments

The authors thank all of the patients at the Chinese People’s Liberation Army General Hospital who formed part of the treatment groups.

## Conflict of interest

The authors declare that the research was conducted in the absence of any commercial or financial relationships that could be construed as a potential conflict of interest.

## Publisher’s note

All claims expressed in this article are solely those of the authors and do not necessarily represent those of their affiliated organizations, or those of the publisher, the editors and the reviewers. Any product that may be evaluated in this article, or claim that may be made by its manufacturer, is not guaranteed or endorsed by the publisher.
