# Supplementary material for: Expression and clinical significance of Cathepsin K and MMPs in invasive non-functioning pituitary adenomas
Source: Front Oncol. 2022 Aug 16;12:901647. doi: 10.3389/fonc.2022.901647 (PMC9424993; doi:10.3389/fonc.2022.901647)
Supplement: Supplementary file 1 [file Table_1.docx]

**Table S1:** tumor size in high-expression and low-expression of MMP9, MMP2, TIMP2 and PTTG1.

|  | High-expression | Low-expression | P |
| --- | --- | --- | --- |
| CTSK | 3.04 ± 0.82 | 2.76 ± 0.69 | **0.028** |
| MMP9 | 2.95 ± 0.74 | 2.95 ± 0.88 | 0.995 |
| MMP2 | 3.03 ± 0.73 | 2.79 ± 0.87 | 0.058 |
| TIMP2 | 2.93 ± 0.81 | 2.98± 0.75 | 0.651 |
| PTTG | 2.99 ± 0.77 | 2.86 ± 0.83 | 0.319 |

Data are shown as mean ± standard deviation (SD)
