# Supplementary material for: Expression and clinical significance of Cathepsin K and MMPs in invasive non-functioning pituitary adenomas
Source: Front Oncol. 2022 Aug 16;12:901647. doi: 10.3389/fonc.2022.901647 (PMC9424993; doi:10.3389/fonc.2022.901647)
Supplement: Supplementary file 2 [file Table_2.docx]

**Table S2:** Relationship between MMP9, MMP2, TIMP2, and PTTG1 expression and clinical features of patients**.**

| Clinical features | N | MMP9 | | P | MMP2 | | P | TIMP2 | | P | PTTG1 | | P |
| --- | --- | --- | --- | --- | --- | --- | --- | --- | --- | --- | --- | --- | --- |
|  |  | High 116 | Low 58 |  | High 116 | Low 58 |  | High 116 | Low 58 |  | High 115 | Low 59 |  |
| Sex |  |  |  |  |  |  |  |  |  |  |  |  |  |
| Male | 86 | 55 | 31 | 0.453 | 63 | 23 | 0.068 | 52 | 34 | 0.130 | 60 | 26 | 0.311 |
| Female | 88 | 61 | 27 |  | 53 | 35 |  | 64 | 24 |  | 55 | 33 |  |
| Age (year) |  |  |  |  |  |  |  |  |  |  |  |  |  |
| <50 | 90 | 62 | 28 | 0.520 | 57 | 33 | 0.334 | 59 | 31 | 0.748 | 56 | 34 | 0.264 |
| ≥50 | 84 | 54 | 30 |  | 59 | 25 |  | 57 | 27 |  | 59 | 25 |  |
| LTD |  |  |  |  |  |  |  |  |  |  |  |  |  |
| ≤3cm | 104 | 70 | 34 | 0.827 | 62 | 42 | **0.016** | 71 | 33 | 0.585 | 66 | 38 | 0.372 |
| >3cm | 70 | 46 | 24 |  | 54 | 16 |  | 45 | 25 |  | 49 | 21 |  |
| Invasion |  |  |  |  |  |  |  |  |  |  |  |  |  |
| IPAs | 94 | 71 | 23 | **0.007** | 76 | 18 | **<0.001** | 56 | 38 | **0.031** | 69 | 25 | **0.027** |
| SS-IPAs | 44 | 26 | 18 | **0.001** | 30 | 14 | **0.003** | 26 | 18 | 0.929 | 31 | 13 | 0.544 |
| CS-IPAs | 50 | 45 | 5 |  | 46 | 4 |  | 30 | 20 |  | 38 | 12 |  |
| NIPAs | 80 | 45 | 35 |  | 40 | 40 |  | 60 | 20 |  | 46 | 34 |  |
| Compression symptoms |  |  |  |  |  |  |  |  |  |  |  |  |  |
| Yes | 112 | 71 | 41 | 0.218 | 71 | 41 | 0.218 | 74 | 38 | 0.823 | 73 | 39 | 0.732 |
| No | 62 | 45 | 17 |  | 45 | 17 |  | 42 | 20 |  | 42 | 20 |  |
| Pituitary apoplexy |  |  |  |  |  |  |  |  |  |  |  |  |  |
| Yes | 30 | 22 | 8 | 0.395 | 21 | 9 | 0.670 | 18 | 12 | 0.395 | 24 | 6 | 0.077 |
| No | 144 | 94 | 50 |  | 95 | 49 |  | 98 | 46 |  | 91 | 53 |  |
| Tumor texture |  |  |  |  |  |  |  |  |  |  |  |  |  |
| Cystic | 7 | 6 | 1 |  | 6 | 1 |  | 5 | 2 |  | 6 | 1 |  |
| Solid | 110 | 77 | 33 | 0.118 | 78 | 32 | 0.056 | 74 | 36 | 0.759 | 74 | 36 | 0.450 |
| Cystic & solid | 57 | 33 | 24 |  | 32 | 25 |  | 37 | 20 |  | 35 | 22 |  |
| Resection degree |  |  |  |  |  |  |  |  |  |  |  |  |  |
| Total | 132 | 86 | 46 | 0.452 | 84 | 48 | 0.133 | 89 | 43 | 0.707 | 91 | 41 | 0.160 |
| Residual | 42 | 30 | 12 |  | 32 | 10 |  | 27 | 15 |  | 24 | 18 |  |
| Recurrence |  |  |  |  |  |  |  |  |  |  |  |  |  |
| Yes | 63 | 41 | 22 | 0.738 | 44 | 19 | 0.503 | 45 | 18 | 0.315 | 43 | 20 | 0.650 |
| No | 111 | 75 | 36 |  | 72 | 39 |  | 71 | 40 |  | 72 | 39 |  |

#: P-value for comparison of IPAs and Non-IPAs, *P value of comparison of SS-IPAs and CS-IPAs, LTD: the largest tumor diameter, SS: sphenoid sinus, CS: cavernous sinus, IPAs: invasive pituitary adenomas, NIPAs: non-invasive pituitary adenoma
